# Gene therapy: principles, challenges and use in clinical practice

**DOI:** 10.1007/s00508-024-02368-8

**Published:** 2024-05-07

**Authors:** Cihan Ay, Andreas Reinisch

**Affiliations:** 1 Department of Medicine I, Clinical Division of Haematology and Haemostaseology, Medical University of Vienna, Währinger Gürtel 18-20, 1090 Vienna, Austria; 2 Department of Medicine, Division of Hematology & Department for Blood Group Serology and Transfusion Medicine, Medical University of Graz, Auenbruggerplatz 38, 8036 Graz, Austria

**Keywords:** Gene therapy, Gene transfer, Hereditary diseases, Hemophilia, Spinal muscular atrophy

## Abstract

**Introduction:**

Gene therapy is an emerging topic in medicine. The first products have already been licensed in the European Union for the treatment of immune deficiency, spinal muscular atrophy, hemophilia, retinal dystrophy, a rare neurotransmitter disorder and some hematological cancers, while many more are being assessed in preclinical and clinical trials.

**Objective:**

The purpose of this review is to provide an overview of the core principles of gene therapy along with information on challenges and risks. Benefits, adverse effects and potential risks are illustrated based on the examples of hemophilia and spinal muscular atrophy.

**Results:**

At present, *in-vitro* and *in-vivo* gene addition or gene augmentation is the most commonly established type of gene therapy. More recently, more sophisticated and precise approaches such as *in situ* gene editing have moved into focus. However, all types of gene therapy require long-term observation of treated patients to ensure safety, efficacy, predictability and durability. Important safety concerns include immune reactions to the vector, the foreign DNA or the new protein resulting from gene therapy, and a remaining low cancer risk based on insertional mutagenesis. Ethical and regulatory issues need to be addressed, and new reimbursement models are called for to ease the financial burden that this new treatment poses for the health care system.

**Conclusion:**

Gene therapy holds great promise for considerable improvement or even cure of genetic diseases with serious clinical consequences. However, a number of questions and issues need to be clarified to ensure broad accessibility of safe and efficacious products.

## Introduction

Already in 1972, Friedmann and Roblin hypothesized that genetic modification might be the way to cure hereditary diseases [1]. Following many years of scientific groundwork and technical advancements, the first clinical gene therapy studies started in the early 1990s [2]. Over the years, several major setbacks, including the tragic death of a patient treated in a gene therapy trial in 1999 and several cases of unintended insertional mutagenesis and development of acute leukemia, slowed the development [3–7]. The 18-year-old patient who died in 1999 had partial ornithine transcarbamoylase (OTC) deficiency, a genetically determined metabolic disorder of the urea cycle, and received an infusion of corrective OTC gene encased in a recombinant adenoviral vector [8]. A severe immune reaction evoked by the adenoviral vector led to his death four days after the administration. This case highlighted the potential of the vector itself to pose a risk, adequate training of the health care staff and the implementation of basic operating procedures, among others.

The lessons learned from these events enabled continuous improvements, and the unprecedented results that are achievable with gene therapy led to the development of a myriad of new products currently tested in clinical trials for a broad range of indications [9]. Today, gene therapies have emerged as promising treatment options and are rapidly entering the treatment landscape of various inherited and acquired diseases including immune disorders, neurodegenerative diseases, hemophilia, ocular diseases, hemoglobinopathies, or cancer. Some gene therapies have already been approved for clinical use, and many more are being developed at increasing speed.

Although continuous collection of additional long-term safety data will be necessary in the future, the growing importance of gene therapy is beyond doubt and calls for appropriate knowledge among health care professionals. There is currently a high unmet need for education, as revealed by a survey conducted among hospital physicians in Austria [10]. To address this knowledge gap, this review summarizes core principles, benefits, potential risks and challenges of gene therapy, with a particular focus on hemophilia and spinal muscular atrophy, and discusses future perspectives.

## Basic principles of gene therapy

### Methods and techniques of gene therapy: gene addition/augmentation vs. gene suppression

Gene therapy is the transfer of genetic material to a patient to treat or potentially even cure a disease. There are various approaches of gene therapy. Most currently used gene therapy products attempt to replace the function of a defective gene with that of a healthy gene. The genetic material (aka transgene) should ideally be delivered to the physiologically relevant target tissue where it is expressed at a physiologically meaningful level and in a stable manner. Interference of the gene or its protein products with the integrity of the target cells must be avoided [11].

**Fig. 1:**
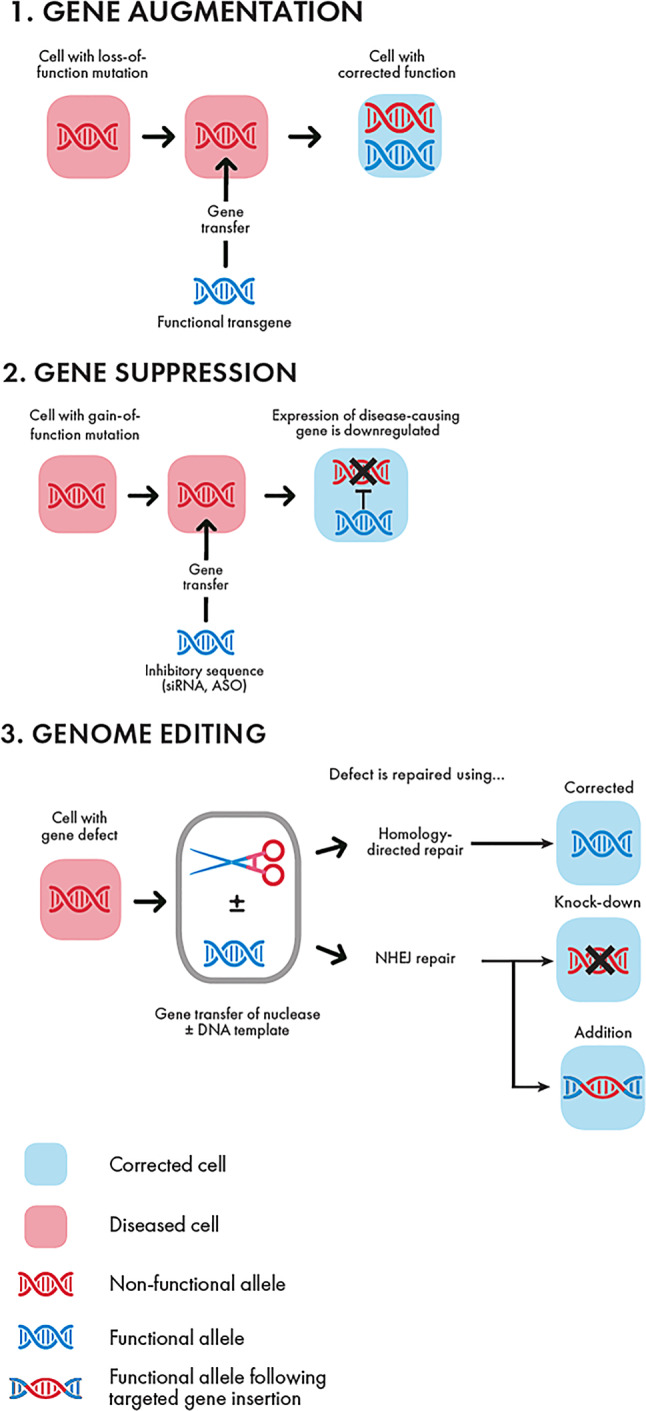
Types of gene therapy: gene augmentation, gene suppression, and genome editing (according to Anguela XM and High KA, and Porteus MH) [2, 12]. Foot note: *NHEJ* non-homologous end joining; repair of double-strand breaks via direct ligation of the break ends without a homologous template

This addition or substitution of genes with loss-of-function defects (Fig. 1a) is called gene addition/augmentation [2]. In case of gene augmentation, the newly transferred functional copy of a gene is present in the cell nucleus together with the defective gene.

A different approach is required when the disease is caused by gain-of-function defects. Suppression of gain of function can be achieved by the import of inhibitory sequences (i.e., microRNAs, short hairpin RNAs) into target cells (Fig. 1b).

### Genome editing

**Fig. 2:**
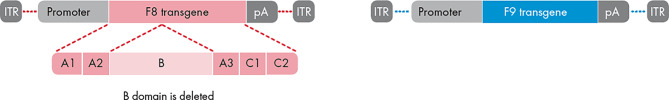
Structure of adeno-associated viral vectors for the treatment of hemophilia (**a**) and (**b**). Foot note: *ITR* inverted terminal repeat; *pA* polyadenylation signal

More recently, with the discovery of novel tools that can precisely target and manipulate DNA, *in situ* repair of genetic defects has become possible. This approach is called genome editing and allows for the correction of genetic defects with single base-pair precision (Fig. 1c; [2, 12]). Genome editing mostly relies on the site-specific introduction of a double-strand break (DSB) into DNA. The nuclease-induced site-specific DSB in the genome stimulates active endogenous repair mechanisms. The two best understood repair mechanisms are non-homologous end-joining (NHEJ) and homology-directed repair (HDR). In NHEJ, the DSB is fixed simply by joining the two ends of the broken DNA. This mechanism is relatively error-prone and frequently leads to insertions and deletions (indels) that can result in the functional loss of a gene. In stark contrast, HDR—the naturally occurring recombination mechanisms observed in mammalian cells—involves the use of the sister chromatide as a template for a “copy and paste” process known as homologous recombination. However, for genome editing, a cell can be tricked, and a DNA donor repair template can be introduced into the cell that will be used for homologous recombination instead of the sister chromatide. Importantly, this DNA repair template can be engineered to correct a mutation or even integrate additional genetic material.

CRISPR-Cas9 is currently the platform that is used most frequently for the introduction of DNA DSBs. However, other nucleases such as zinc-finger nucleases, transcription activator-like effector nucleases and homing endonucleases-meganucleases offer comparable precision.

Genome editing can be performed in cells both outside and inside of the body (*ex vivo* and *in vivo*, see below) [12]. Clinical trials are currently assessing the utility of genome editing in the correction of monogenic diseases and cell-based regenerative medicine; also, enhancement of chimeric antigen receptor (CAR) T cell therapy is investigated. In principle, genome editing has the potential to halt the progression of most monogenic diseases, provided that patients are treated before irreversible damage has occurred. The first CRISPR-based gene therapy, which has been approved only recently in the European Union is exagamglogene autotemcel for the treatment of transfusion-dependent β‑thalassemia and severe sickle cell disease [13].

### Methods of gene transfer: ex vivo vs. in vivo gene therapy

*Ex vivo* gene therapy requires the extraction of cells from the patient [14]. After the successful introduction of a transgene or successful gene editing, the cells are re-administered to the patient. *Ex vivo* gene therapies commonly use cells of hematopoietic origin such as hematopoietic stem cells or T cells, and are being developed for the treatment of inherited immune disorders, neurodegenerative diseases (e.g., X‑linked adrenoleukodystrophy, metachromatic leukodystrophy), β‑hemoglobinopathies (e.g., β‑thalassemia, sickle cell disease), and cancer.

Today, gene therapy for cancer has mainly been established in the form of CAR T cell therapy. CARs endow T cells with the ability to target antigens expressed on the surface of tumor cells. CD19, an antigen present in most B cell malignancies, constitutes the classical target, with several products having been approved to date. However, CAR T cell applications are extending to other hematologic malignancies as well as solid tumors.

In contrast to *ex vivo* gene therapy, *in vivo* gene therapy relies on the administration of a vector that carries and delivers the transgene to a target tissue (e.g., liver, neurons, muscle). Most current *in vivo* gene therapies target the patient’s liver and can be applied intravenously [15]. *In vivo* gene therapy usually does not require conditioning prior to administration; thus, prolonged hospital stays are avoided, and the treatment can frequently even be conducted in the outpatient setting. Moreover, this approach is attractive as it dispenses with the need for elaborate steps involved in *ex vivo* treatment including cell collection from the patient and manipulation in a specialized facility before re-administration [16]. However, the feasibility of *in vivo* administration depends on tissue-specific targeting or local delivery and/or target-cell-specific gene expression.

*In vivo* gene therapy primarily focuses on rare monogenic disorders caused by loss-of-function or toxic gain-of-function mutations. Among others, *in vivo* gene therapy is currently being evaluated or has already been approved in the treatment of hemophilia A and B, neuromuscular disorders (e.g., spinal muscular atrophy, Duchenne muscular dystrophy) and various types of inherited blindness (e.g., *RPE65* mutation-associated retinal dystrophy, achromatopsia, choroideremia, Leber’s hereditary optic neuropathy, X‑linked retinoschisis, and X-linked retinitis pigmentosa). In the setting of central nervous system diseases, targeting a sufficient number of cells to achieve an adequate level of gene modification is challenging [2].

### Vectors

Vectors are vehicles that can carry genetic material and introduce it into target cells. For gene transfer, mainly naturally occurring viruses are genetically modified for the purpose of transferring and expressing a transgene. In viral vectors, the viral genome is replaced by the gene therapy transgene. One fundamental distinction between the viruses used for gene therapy is their inherent capacity to integrate into the host DNA. Therefore, viral vectors can broadly be classified as either integrating or non-integrating [17, 18].

Integrating viral vectors are introduced into cells with the aim of stably incorporating therapeutic genes into the genome, thus allowing the cells to pass the transgene onto every daughter cell. These vectors, which are typically derived from retro- and lentiviruses, are frequently employed for *ex vivo *gene therapy.

With non-integrating viral vectors, on the other hand, the transferred DNA is stabilized extrachromosomally as an episome. Since the transgene is usually not integrated into the genome, it needs to be delivered to long-lived, non-dividing, post-mitotic cells where it will be expressed for the life of the target cell only. Episomes are stable in non-dividing cells for long periods and provide sustained transgene expression [19, 20]. On the downside, transgene expression may be lost over time upon cell proliferation due to the lack of vector genome replication with cell division [21]. Non-integrating vectors are typically used for *in vivo *gene therapy.

Recombinant adeno-associated viral (rAAV) vectors have emerged as the platform of choice for *in vivo* gene therapies due to their advantages of relatively low immunogenicity, targeted gene delivery into a range of tissues, and long-term expression of the transgene [22–24]. AAV is a very small (*parvovirus)* single-stranded DNA virus that is non-pathogenic and naturally replication-defective. Wild-type AAV requires the presence of another virus, such as an adenovirus, to replicate [25]. In the process of engineering, all viral coding sequences including the *rep *and *cap *genes that are responsible for replication and the structure of the viral capsid are replaced with a gene expression cassette of interest. This includes not only the therapeutic gene but also other transcriptional regulatory elements such as a promoter sequence that facilitates transgene expression within specific cell types [26]. rAAV vectors have tropism for specific tissues depending on their serotype, with serotypes ranging from AAV1 to AAV13 [27]. Features of different vectors are discussed in Table 1 [2, 28].

**Table 1:** Most commonly used vectors in gene therapy (according to Anguela XM & High KA, and Lundstrom K) [2, 28]

Features	Retroviral	Lentiviral	Adenoviral	Adeno-associated viral	HSV‑1
Viral genome	ssRNA	ssRNA	dsDNA	ssDNA	dsDNA
Cell type	Dividing	G1 phase	Dividing and non-dividing	Non-dividing	Non-dividing
Transgene sizes	8 kb	8 kb	8–30 kb	4.5 kb	50–130 kb
Integration potential	Yes	Yes	Poor	Poor	Poor
Route of application	Ex vivo	Ex vivo	In vivo	In vivo	In vivo/topical
Long-term expression	Yes	Yes	No	Yes	Yes
Advantages & Limitations	Persistent gene expression in dividing cells due to integration, low immune response	Persistent gene expression in dividing cells due to integration, low immune response	High cell tropism with strong immunogenicity, transduction limited to non-dividing cells for persistent expression	Low immunogenicity, non-human pathogenic, transduction limited to non-dividing cells for persistent expression	High cell tropism, transduction limited to non-dividing cells for persistent expression

*ss* single-stranded; *ds* double-stranded

For the large-scale production of rAAV vectors, platforms based on human embryonic kidney cells (HEK) or the insect cell line Spodoptera frugiperda (SF9) with recombinant baculoviruses have been widely employed [22]. rAAVs are administered via a single infusion either intravenously or locally. If given intravenously, the vector will transduce the target cell depending on its tissue tropism. Once bound to the cell via receptors, the virus gets endocytosed. After escaping from the endosome, rAAV particles enter the cell nucleus, the viral capsid gets uncoated, and after a second strand synthesis of the transgene, the host cell’s endogenous transcription and translation machinery is used for the production of a functional protein. Based on the fact that rAAVs integrate into the host genome at very low frequencies, rAAV is considered to bear only low risk of genotoxicity [29–33]. In clinical studies investigating valoctocogene roxaparvovec and etranacogene dezaparvovec that have been licensed for the treatment of hemophilia A and B, respectively, transgene DNA was temporarily detected in semen; therefore, barrier contraception is recommended for 6 and 12 months after the administration of valoctocogene roxaparvovec and etranacogene dezaparvovec, respectively, in patients with reproductive potential [34, 35]. Moreover, treated patients should not donate semen, blood, organs, tissues or cells for transplantation.

## Risks of gene therapy

Depending on the type of gene therapy (*ex vivo* vs. *in vivo*, integrating vs. non-integrating vectors), several safety-related issues need to be taken into consideration and should be discussed with the potential patient.

Integrating vectors such as retro- and lentiviruses that are primarily used for *ex vivo* gene therapy bear the risk of insertional mutagenesis due to their semi-random integration into the DNA. This can potentially induce the activation of an oncogene or the disruption of a tumor suppressor gene, thereby leading to the formation of cancer [6, 36–38]. Unfortunately, T cell leukemia developed in some of the early trials using γ‑retroviral vectors for severe combined immunodeficiencies (SCID) [39]. Over time, the risk of insertional mutagenesis has been reduced by the development of safer vectors [18]. Compared to γ‑retroviral vectors, lentiviral vectors have a safer integration pattern and higher transduction efficiencies. However, clinical-scale production of lentiviral vectors is challenging. Nevertheless, specific surveillance and long-term follow-up is necessary. In the future, such unintentional detrimental integration events might be avoided by using the very precise genome editing technology [18].

In contrast to integrating viral vectors that are primarily used for *ex vivo* gene therapy applications, non-integrating viral vectors are mainly used for *in vivo* gene therapy. These have only minimal rates of integration into the donor DNA and consequently confer a very low probability of causing insertional mutagenesis and cancer. Hemophilic dogs treated with AAV gene therapy had low but detectable levels of AAV integration into the genomic DNA and did not show any evidence of tumor formation after 10 years of follow-up [40]. Studies in neonatal mice implicated that pathogenic AAV integration events might actively contribute to hepatocellular cancer development, although potential genotoxic events are highly dependent on factors including AAV integration preferences, vector design, vector dose and, in particular, recipient age at AAV injection [41–43].

Since non-integrating vectors are applied *in vivo*, they carry the risk of evoking immune responses that are potentially life-threatening or might impair the long-term efficacy of treatment. Immune responses and related adverse events seem to be directly associated with the vector doses applied [44, 45]. Uncontrolled immune responses are the main culprit with regard to most severe adverse events linked to AAV gene transfer, including fatal hepatotoxicity, dorsal root ganglia toxicity, and myocarditis. Notably, the human body contains immune-privileged sites (e.g., the central nervous system) and immunosuppressive microenvironments (e.g., the liver) where AAV vectors are less likely to trigger strong responses than at other sites such as the circulation or the muscle [46].

Uncontrolled innate immunes responses such as overactivation of the complement pathway with subsequent induction of thrombotic microangiopathy have been described following AAV gene therapy. Thrombotic microangiopathy is a hematologic emergency situation caused by microscopic blood clots in the capillaries and small blood vessels, leading to organ damage, anemia and low platelet counts [47]. Also, the adaptive immune system can cause dangerous side effects via CD8+ cytotoxic T‑cell responses, such as T‑cell mediated hepatotoxicities associated with inflammatory reactions that have been observed in AAV9 vector therapy for spinal muscular atrophy.

Immune responses to vectors can be mitigated by the administration of immunomodulatory drugs such as corticosteroids [18]. However, immune system-mediated toxicity is still a challenge for successful gene transfer using AAV vectors, particularly in settings in which the treatment of the targeted genetic disease requires high doses [48].

## Approved therapies and fields of investigation

A number of gene therapy products have been licensed over the last seven years in Europe, the United States and other countries. Currently, a total of six CAR T cell products have received approval in Europe. Tisagenlecleucel, axicabtagene ciloleucel, brexucabtagene autoleucel and lisocabtagene maraleucel are used for the treatment of patients with B‑cell malignancies (e.g., lymphoma); all of these target the CD19 antigen [49–52]. Tisagenlecleucel is also indicated for acute lymphoblastic leukemia [49]. The BCMA-directed therapies idecabtagene vicleucel and ciltacabtagene autoleucel have been licensed for the treatment of multiple myeloma [53, 54]. Talimogene laherparepvec is a modified oncolytic herpes virus that is used as an intralesional cancer immunotherapy for advanced melanoma [55].

In addition, at the time of the publication of this review, gene therapies are available in Europe for serious monogenic disorders including severe combined immunodeficiency due to adenosine deaminase deficiency (ADA-SCID; autologous CD34+ cells transduced with a retroviral vector that encodes for the human ADA complementary DNA sequence), biallelic *RPE65* mutation-associated retinal dystrophy (voretigene neparvovec), aromatic L‑amino acid decarboxylase (AADC) deficiency (eladocagene exuparvovec), metachromatic leukodystrophy (atidarsagene autotemcel), spinal muscular atrophy (onasemnogene abeparvovec), hemophilia A (valoctocogene roxaparvovec) and B (etranacogene dezaparvovec) and β‑thalassemia as well as sickle cell disease [13, 34, 35, 56–60].

A multitude of trial programs is currently evaluating gene therapies in a broad range of diseases. Approximately 1500 products are being tested in the pre-clinical setting and in more than 500 clinical studies. In addition to the mentioned indications, gene therapy is being assessed in inherited metabolic diseases such as ornithine transcarbamylase deficiency (NCT02991144), homozygous familial hypercholesterolemia (NCT02651675) and mucopolysaccharidosis type VI (NCT03173521), in age-related macular degeneration (NCT01024998, NCT01301443, NCT01494805, NCT03066258) and previously untreatable disorders like Huntington’s disease (NCT03761849), among many others. As an example, achievements and limitations of established gene therapies are delineated below for hemophilia and spinal muscular atrophy.

## Hemophilia a and b

Hemophilia, an X‑linked recessive bleeding disorder, is caused by a deficiency of coagulation factor VIII (hemophilia A) or IX (hemophilia B) due to mutations in the genes encoding for these factors. Several characteristics make hemophilia A and B an ideal target for gene therapy: this is a monogenic, recessive disease which results in a large range of affected protein levels [61–63]. Moreover, the bleeding phenotype is responsive to increases of factor levels, and their measurement provides monitoring of the treatment efficacy. While FVIII is synthesized in the sinusoidal endothelial cells of the liver, FIX synthesis takes place in the hepatocytes [63, 64]. The majority of defects of the *F8* gene are caused by intron 22 inversions; in the *F9* gene, missense mutations are mainly responsible for the absence or dysfunction of the clotting factor [65, 66].

In patients with hemophilia, FVIII or FIX deficiency leads to bleeding into joints, muscles and soft tissues, eventually giving rise to joint damage, disability and chronic pain as the most common consequences [61]. The traditional treatment consists of intravenous replacement of coagulation factor concentrates at regular intervals, given the relatively short half-life of these factors. This puts a considerable burden on patients and care givers. Furthermore, persons with hemophilia may develop inhibitory antibodies that diminish the efficacy of factor replacement. Despite regular prophylaxis, the risk of arthropathy is not completely reduced with the current treatment options. Moreover, the treatment confers a significant cost burden, and access to factor products is limited in many countries.

Valoctocogene roxaparvovec was the first gene therapy to be licensed for the treatment of hemophilia A and became available in August 2022 in the European Union [34]. Similarly, etranacogene dezaparvovec was approved as the first gene therapy for hemophilia B in February 2023 [35].

Gene therapy for hemophilia is liver-directed as the vectors target hepatocytes, which act as protein factories to release the transgene product into the circulation. AAV vectors with the serotype 5 are used for both currently approved liver-directed therapies. This treatment is expected to transform severe disease phenotypes into mild or normal phenotypes based on sustained elevation of clotting factor levels [2, 63, 67, 68]. The continuous expression of coagulation factors provides protection from bleeding, renders prophylaxis at regular intervals unnecessary and contributes to increased quality of life.

While hemophilia A and B show similar clinical symptoms, their molecular bases differ. As FVIII complementary DNA is larger than FIX complementary DNA (approximately 9 kb vs. 1.5 kb), modification is required to enable packaging of the *F8* transgene into the recombinant AAV5 (rAAV5) vector [65, 66, 69] that has limited packaging capacity of approximately 4.7 kb (Fig. 2). To fit the *F8* transgene into AAV, the large B‑domain of *F8* is deleted, resulting in a length of approximately 5 kb. For the *F9* transgene, a naturally occurring but more active variant of FIX that was initially described in a family in Padua (i.e., the Padua variant) is often used [70]. The therapy is administered as a single intravenous infusion, with dosing based on body weight. Following the administration, patients may develop a mild viral syndrome consisting of transient fever, myalgia, and malaise [71–73].

AAVs naturally infect humans, and upon infection the human immune system develops neutralizing antibodies that are a particular challenge for AAV-based gene therapy approaches. These pre-existing neutralizing anti-AAV antibodies impede gene transfer by inhibiting the transduction of target cells by the AAV-based vector [74]. Measurable antibodies to different AAV serotypes have been found in approximately 30–60% of the population [75]. Prior to treatment with the gene therapy product approved for hemophilia, the levels of neutralizing antibodies need to be assessed. Only patients without antibodies according to a validated assay are eligible for the administration of valoctocogene roxaparvovec [34]. With respect to etranacogene dezaparvovec, patients with pre-existing anti-AAV5 antibodies were not excluded from the phase III trial. Trials results showed that gene therapy can be successful even in the presence of low titers of pre-existing neutralizing antibodies; however, the titer should not exceed 1:678 according to the specific assay employed for etranacogene dezaparvovec [35].

Accurate and robust detection of neutralizing anti-AAV antibodies is important but not easy to achieve as the required assays have not been established in clinical routine yet. Furthermore, no universal method has been implemented to reliably measure the amount of clinically relevant antibody levels [76]. Transduction inhibition assays and total antibody assays are used, although meaningful comparisons across assays are nearly impossible due to the lack of standardization. The limited availability of head-to-head studies that align assay results with clinical outcomes renders the interpretation and implementation of screening titer cutoffs difficult [77].

Another issue that requires attention is the emergence of potential immune responses against capsid proteins or even the transgene and its products that can lead to rejection of the transduced cells [78–80]. In a high number of patients, liver-directed gene therapy for hemophilia led to modest increases in the liver transaminases alanine aminotransferase (ALT) and aspartate aminotransferase (AST) [78]. Although all hemophilia gene therapy clinical trials have shown transaminitis, this was more frequently seen in patients receiving hemophilia A gene therapy than in those undergoing hemophilia B gene therapy [81, 82]. In the majority of cases, the reported elevations in ALT levels showed a 1.5- to 2‑fold peak above the upper limit of normal. Unfortunately, the mechanisms responsible for ALT elevation potentially reflecting liver damage have not been fully unraveled to date, but cytotoxic T‑cell attacks against transduced cells and/or cellular stress induced by the accumulation of misfolded protein in the endoplasmic reticulum are suspected [20, 23, 83]. Transaminitis mainly occurred within the first 12 weeks after vector infusion and either preceded a loss of transgene expression or coincided with it [78, 84]. In clinical studies, immunosuppression with corticosteroids was initiated with the aim of dampening the immune system and thereby preserving the expression of the gene therapy product [80].

During the first weeks and months following administration of gene therapy, close clinical and laboratory monitoring is mandatory [66]. If the gene transfer is successful, the need for exogenous administration of coagulation factor products generally declines considerably until the endogenous factor production has sufficiently increased to render factor replacement therapy unnecessary [85, 86]. However, patients need to be aware of considerable inter-patient outcome variability that has been observed in clinical trials. Additionally, each gene therapy product has its unique features, including vector design and vector dose, AAV serotype and the production platform used for manufacturing. Patient variables include previous AAV exposure, patient-specific antigen processing, and hepatic health prior to gene therapy [44, 71, 85, 87–89]. Further research and long-term observation is needed to gain additional insights, especially with respect to safety as well as predictability and durability of factor expression.

## Spinal muscular atrophy

Loss-of-function mutations in the survival motor neuron 1 (*SMN1*) gene give rise to spinal muscular atrophy (SMA) [90]. This autosomal recessive disease is characterized by the degeneration of alpha motor neurons located in the spinal cord. Progressive muscle weakness, paralysis, loss of bulbar function and death from respiratory complications occur at around 2 years of age in most patients [91, 92]. Infantile-onset (type 1) SMA is the most severe and most common subtype of SMA [93]. It usually manifests before the age of 6 months and is the most common genetic cause of death in infants; however, symptoms may already be present at birth.

The antisense oligonucleotide drug nusinersen has revolutionized the treatment of patients with SMA. It targets the *SMN2* gene, which is a nearly identical copy of the *SMN1* gene, and produces functional SMN protein, although only a fraction of the amount obtained from the intact *SMN1* gene, and thus cannot compensate for the loss of *SMN1 *[94, 95]. By modulating alternative mRNA splicing of the *SMN2* gene in spinal motor neurons, nusinersen induces higher expression of *SMN2*, thereby better compensating for the *SMN1* loss. However, this treatment involves repeated intrathecal administration (i.e., direct injection into cerebrospinal fluid) with up to seven injections during the first year followed by maintenance doses every 4 months. The treatment costs are substantial, and patients with advanced disease still rely on assisted respiration using non-invasive ventilation [96]. In addition, the oral SMN2 pre-mRNA splicing modifier risdiplam has been approved for the treatment of patients with 5q-autosomal recessive SMA with a clinical diagnosis of SMA types 1, 2, or 3, or with one to four copies of the *SMN2* gene [97].

The first gene therapy for patients with SMA is onasemnogene abeparvovec, which was approved in the European Union in 2020. It is indicated in patients with SMA linked to chromosome 5q, a biallelic mutation in the *SMN1* gene and clinically apparent SMA type 1, or 5q-associated SMA with a biallelic mutation in the *SMN1* gene and up to three copies of the *SMN2 *gene [60]. Onasemnogene abeparvovec is an AAV-based gene therapy that is administered as a one-time intravenous infusion. The AAV vector serotype 9 (AAV9) delivers a fully functional copy of the *SMN* gene into the target motor neuron cells, leading to expression of the SMN protein.

SMA patients treated with onasemnogene abeparvovec show improvements in muscle movement and function, significant improvement in their ability to reach developmental motor milestones, and survival prolongation. Long-term study results suggest evidence of sustained clinical efficacy. The phase I START study included symptomatic infants with SMA type 1 and two copies of the *SMN2* gene [98]. After a median of 5.2 years post gene therapy, all 10 patients in the therapeutic-dose cohort remained alive and without the need for permanent ventilation. All of them had maintained previously acquired motor milestones, and two had achieved the milestone of standing without assistance. The phase III SPR1NT trial evaluated onasemnogene abeparvovec in pre-symptomatic children with biallelic *SMN1 *mutations treated within 6 weeks after birth [99]. Among 15 children with three *SMN2* copies, all were able to stand independently before 24 months, and 14 walked independently. All of them survived without permanent ventilation at 14 months.

Mandatory assessments prior to the treatment include measurement of pre-existing AAV9 antibodies using a validated assay and liver function tests. The most common side effects of onasemnogene abeparvovec comprise elevation of liver enzymes and vomiting. As acute hepatic failure with a fatal outcome has been reported, it is recommended to monitor the liver function regularly for at least 3 months after treatment [100]. Immune responses to the vector are assumed to be the cause of hepatotoxicity; therefore, a prophylactic corticosteroid regimen needs to be administered. Moreover, available data suggest that overexpression of the SMN protein, especially in the sensorimotor circuit, might lead to gain of toxic function [101]. A long-term follow-up study (NCT03421977) of the completed phase 1 study (NCT02122952) is assessing safety for up to 15 years, with final results expected for December 2033.

## Challenges & perspectives

Gene therapy has opened new doors in the treatment of a range of serious and debilitating diseases. However, many remaining challenges need to be fully addressed before gene therapy can become a routine treatment for monogenic diseases [18, 102]. These include mainly aspects related to safety, predictability, and the durability of the gene therapy outcomes. For *in vivo* gene therapy, better understanding of immune responses is needed, and systematic long-term efficacy and safety assessment of every treated patient will be essential. Moreover, manufacturing and regulatory challenges need to be solved to make gene therapies broadly accessible. Since gene therapy requires well-trained personnel working at specialized facilities, the number of centers providing these therapies will be limited [2].

Finally, a societal consensus needs to be reached regarding disputed issues such as the very high financial burden [18]. One-time gene therapies tend to be extremely expensive up-front, although cost-benefit analyses that take patient quality of life and lifelong medical costs of currently available treatments into account may provide justification for the use of gene therapy products [103]. In addition, treatment options have been completely absent for a range of serious diseases to date. Nevertheless, keeping the expenses at a reasonable level will be important to improve equality of access. Dedicated funding programs can help to lower the financial burden. Negotiations with health insurances and government agencies might result in the development of new models for reimbursement.

Finally, to implement gene therapy in clinical practice special logistics and a multidisciplinary approach will be required. Various organization in the field of hemophilia propose new delivery models, such as the hub and spoke model to gain access to gene therapy for patients (summarized in [104]). In such a care delivery model a close collaboration and communication between the hub center, which is responsible for administration of gene therapy and the spoke center (i.e. the referral center) is needed to cover the management of the complex patient journey form initial discussion to long-term follow up.

## Conclusion

Gene therapies are promising and offer enormous potential with respect to finally achieving cure of many serious hereditary and non-hereditary diseases. In 2024, the history of their development already spans decades, although in clinical terms, it appears that the journey has barely begun. Study results obtained with approved gene therapies have proven the principle of gene therapy for clinical use. Nevertheless, to make this new treatment approach broadly available, very demanding challenges regarding both medical and regulatory/financial issues need to be addressed. Long-term safety, clinical efficacy and advantages over standard treatment options must be clearly demonstrated to justify the high-cost burden. Also, ethical discussions are needed to determine an acceptable framework for these new procedures. With numerous trials investigating gene therapies in various indications ongoing, patients with devastating diseases can now hope for new and unprecedented treatment options.
